# Distinct Patterns of Smooth Muscle Phenotypic Modulation in Thoracic and Abdominal Aortic Aneurysms

**DOI:** 10.3390/jcdd11110349

**Published:** 2024-11-01

**Authors:** Chien-Jung Lin, Campbell Keating, Robyn Roth, Yasar Caliskan, Mustafa Nazzal, Vernat Exil, Richard DiPaolo, Divya Ratan Verma, Kishore Harjai, Mohamed Zayed, Chieh-Yu Lin, Robert P. Mecham, Ajay K. Jain

**Affiliations:** 1 Division of Cardiology, Department of Internal Medicine, SSM-Saint Louis University Hospital, St. Louis, MO 63110, USA; 2 Department of Cell Biology and Physiology, Washington University School of Medicine, St. Louis, MO 63110, USA; 3 Department of Pediatrics, Washington University School of Medicine, St. Louis, MO 63110, USA; 4 Division of Nephrology and Hypertension, Department of Internal Medicine, SSM-Saint Louis University Hospital, St. Louis, MO 63110, USA; 5 Department of Surgery, SSM-Saint Louis University Hospital, St. Louis, MO 63110, USA; 6 Division of Cardiology, Department of Pediatrics, SSM-Cardinal Glennon Children’s Hospital, St. Louis, MO 63104, USA; 7 Department of Molecular Microbiology and Immunology, Saint Louis University, St. Louis, MO 63104, USA; 8 Department of Surgery, Washington University School of Medicine, St. Louis, MO 63110, USA; 9 Department of Radiology, Washington University School of Medicine, St. Louis, MO 63110, USA; 10 Department of Molecular Cell Biology, Washington University School of Medicine, St. Louis, MO 63110, USA; 11 Department of Biomedical Engineering, Washington University School of Medicine, St. Louis, MO 63110, USA; 12 Department of Pathology and Immunology, Washington University School of Medicine, St. Louis, MO 63110, USA; 13 Division of Gastroenterology and Hepatology, Department of Pediatrics, SSM-Cardinal Glennon Children’s Hospital, St. Louis, MO 63104, USA

**Keywords:** aortic aneurysm, smooth muscle cell, single-cell RNA sequencing

## Abstract

Thoracic and abdominal aortic aneurysms (TAAs and AAAs, respectively) share morphological features but have distinct clinical and hereditary characteristics. Studies using bulk RNA comparisons revealed distinct patterns of gene expression in human TAA and AAA tissues. However, given the summative nature of bulk RNA studies, these findings represent the totality of gene expression without regards to the differences in cellular composition. Single-cell RNA sequencing provides an opportunity to interrogate cell-type-specific transcriptomes. Single cell RNA sequencing datasets from mouse TAA (GSE153534) and AAA (GSE164678 and GSE152583) with respective controls were obtained from the Gene Expression Omnibus. Bioinformatic analysis was performed with the Seurat 4, clusterProfiler, and Connectome software packages (V1.0.1). Immunostaining was performed with standard protocols. Within normal and aneurysmal aortae, three unique populations of cells that express smooth muscle cell (SMC) markers were identified (SMC1, SMC2, and SMCmod). A greater proportion of TAA SMCs clustered as a unique population, SMCmod, relative to the AAA SMCs (38% vs. 10–12%). These cells exhibited transcriptional features distinct from other SMCs, which were characterized by *Igfbp2* and *Tnfrsf11b* expression. Genes upregulated in TAA SMCs were enriched for the Reactome terms “extracellular matrix organization” and “insulin-like growth factor (IGF) transport and uptake by IGF binding proteins (IGFBPs)”, indicating a role for *Igfbp2* in TAA pathogenesis. Regulon analysis revealed transcription factors enriched in TAAs and AAAs. Validating these mouse bioinformatic findings, immunostaining demonstrated that both IGFBP2 and TNFRSF11B proteins increased in human TAAs compared to AAAs. These results highlight the unique cellular composition and transcriptional signature of SMCs in TAAs and AAAs. Future studies are needed to reveal the pathogenetic pathways of *IGFBP2* and *TNFRSF11B*.

## 1. Introduction

Thoracic and abdominal aortic aneurysms (TAAs and AAAs, respectively) share morphological features but have distinct genetic and pathogenetic mechanisms. Genetically, AAAs share risk factors with atherosclerotic cardiovascular diseases, whereas TAAs are less associated with atherosclerosis but exhibit strong heritability [1]. Histologically, both exhibit features of extracellular matrix fragmentation and smooth muscle cell (SMC) loss [2], although AAAs are more prone to have inflammatory cell infiltration and atherosclerosis [3]. Consistent with these findings, prior studies using bulk RNA comparisons revealed distinct patterns of gene expression in human TAA and AAA tissues [4]. However, given the summative nature of bulk RNA studies, these findings represent the totality of gene expression without regards to the differences in cellular composition between TAAs and AAAs, underscoring a knowledge gap.

SMCs are intimately involved in the pathogenesis of aortic aneurysms. The principal functions of SMCs during normal homeostasis are to regulate vascular tone and diameter via contraction and relaxation and to produce the extracellular matrix of the blood vessel. SMCs retain plasticity postnatally; their gene expression and behavior can be modulated with respect to changes in local environment and stress. Broadly, two SMC cell states have been described: a “mature”, “contractile” state and an “embryonic”, “synthetic” state. SMC phenotypic modulation, i.e., shifting from a “contractile” to a “synthetic” phenotype, is classically defined as downregulation of genes involved in SMC contraction and upregulation of genes involved in proliferation, migration, and matrix degradation and is observed in conjunction with multiple vascular pathologies, including aneurysms [5]. Conversely, selective genetic manipulation in SMC results in aortic aneurysms with SMC phenotypic changes [5,6], highlighting the importance of this process in aortic aneurysm formation.

It remains unclear whether SMC phenotypic modulation differs among different aortopathies or different anatomical locations. In order to characterize this further, we performed a meta-analysis of single cell sequencing studies and identified a novel difference of SMC phenotypes between TAAs and AAAs.

## 2. Materials and Methods

### 2.1. Data Source

Single-cell RNA sequencing datasets from murine TAAs and AAAs with respective controls were obtained from the Gene Expression Omnibus (GEO). Data included are listed in Table 1.

### 2.2. Single-Cell RNA Sequencing Data Analysis

Individual datasets were imported into Seurat v4.0. A quality control threshold of gene number > 500 and <5000, with mitochondrial genes representing <10% of the total was chosen after inspecting individual datasets. Principal component analysis (PCA) was used to integrate the datasets after they were normalized (NormalizeData) and the variable features were identified (FindVariableFeatures, “vst” method). Features were selected that are repeatedly variable across datasets (SelectIntegrationFeatures), then each dataset was scaled (ScaleData), and a PCA run (RunPCA) based on these features. Anchors were identified (FindIntegrationAnchors, PCA method) based on which of the datasets were integrated. The standard Seurat workflow was then used for clustering of the integrated dataset using ScaleData, RunPCA, RunUMAP (PCA method, PCs 1–30), FindNeighbors (PCA method, dimension = 30), and FindClusters (resolution = 0.7).

Marker genes for each integrated cluster were determined with FindAllMarkers using the Wilcoxon rank–sum test on the RNA assay. Differential gene expression between groups for each cluster was determined using FindMarkers. Genes expressed in a minimum of 25% of cells in a group and a log-fold change threshold of 0.25 were selected. DotPlot and VlnPlot methods were used for visualization.

Pathway enrichment analysis was performed with the ReactomePA (v1.44.0) and clusterProfiler (v4.8.2) packages using enrichPathway and visualized with compareCluster. Genes with at least a 2-fold (log-fold change > 0.3) were included in the analysis. *p* values were derived from a hypergeometric test and adjusted using a Benjamini–Hochberg procedure. An adjusted *p* value of <0.05 was considered significant. Data were visualized using the dotplot and cnetplot functions.

For transcription factor regulon analysis, transcription factor activity was performed with the VIPER (v1.34.0) and DoRothEA (v1.12.0) R packages [10]. Regulons with a high confidence level (A and B) were chosen for the analysis. The 20 most variable transcription factors per SMC cluster were chosen for supervised clustering with respective to cluster and visualized with the pheatmap function.

For ligand–receptor analysis, the Connectome (v1.0.1) package was used [11]. Ligands and receptor pairs that were both upregulated were chosen and plotted.

The code used for the analysis is available upon request.

### 2.3. Human Thoracic and Abdominal Aortic Aneurysm Samples

Formalin-fixed, paraffin-embedded human aortic aneurysm slides were obtained from the Department of Pathology and Immunology and the Vascular Surgery BioBank at Washington University School of Medicine. Patient demographics are as listed in Table 2. The protocols for collecting human tissue samples were obtained from consenting patients undergoing surgical intervention. The study was approved by the Institutional Review Board of Washington University School of Medicine. Measures were taken to ensure this study complies with the Declaration of Helsinki.

### 2.4. Histology and Immunostaining

Paraffin sections were rehydrated and pressure-cooked in antigen retrieval solution (10 mM Tris, 1 mM EDTA, pH 9) for 10 min. After cooling, the slides were quenched with 0.3% H_2_O_2_, washed in phosphate-buffered saline (PBS), blocked with 2% donkey serum (Sigma-Aldrich, St. Louis, MO, USA) in PBS, and incubated with the primary antibody (mouse anti-TNFRSF11B [5 ug/mL], clone 98A1071, Novus Biologicals; rabbit anti-IGFBP2 [3 ug/mL], EPR18012-257, Abcam, Cambridge, UK) overnight at 4 °C in the blocking solution. Visualization for immunohistochemistry was accomplished using a biotin-conjugated secondary antibody and the streptavidin-based ABC Kits (Vector Labs, Newark, CA, USA) following the manufacturer’s instructions. Nuclei were counterstained with hematoxylin. Slides were dehydrated and mounted with Permount solution (Thermo Fisher, Waltham, MA, USA). A dilution of 1:200 was used for primary antibodies and 1:300 for secondary antibodies.

## 3. Results

### 3.1. Single Cell Sequencing Meta-Analysis of Mouse Ascending and Abdominal Aortic Aneurysms

To understand smooth muscle cell phenotypes in aortic aneurysmal diseases, we queried the GEO for single-cell RNA sequencing data of mouse thoracic ascending and abdominal aortic aneurysms (TAAs and AAAs, respectively). For mouse TAAs, data were obtained from datasets in the GSE153534 series [7], which used the *Fbn1^C1041G/+^* genetic model. We chose to include the dataset with a more advanced disease state (male mice at 24 weeks of age) in the analysis in order to enrich for important differences between the two disease states. Because the AAA datasets below used male mice, we included only the dataset from male *Fbn1^C1041G/+^* mice. For mouse AAAs, datasets from GSE164678 and GSE152583 were included, which involved surgical models of the peri-vascular application of CaCl_2_ and elastase, respectively [8,9]. We chose to include 14-day but not 7-day elastase-treated mice for a more pronounced phenotype. To minimize inherent cell differences due to a hypercholesteremic state, we did not include mouse AAA datasets from *ApoE*^−/−^ with angiotensin models (e.g., GSE118237). The datasets included are summarized in Table 1.

**Figure 1:**
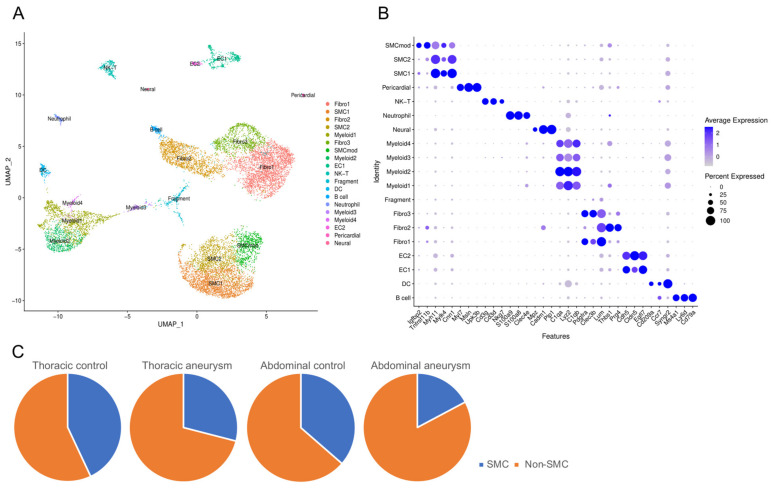
(**A**) UMAP projection of 19 cell types identified after combining mouse TAA and AAA single-cell sequencing datasets. (**B**) Specific gene markers for each cluster. (**C**) Pie graph showing the proportion of SMC (blue) and non-SMC cells (orange) in each condition. Note the reduced proportion of SMCs (blue) in aneurysmal aortae.

The raw data were preprocessed for quality control and integrated for analysis using Seurat v4. As shown in Figure S1, CaCl_2_ and elastase treatment yielded similar cell type distributions; these datasets were combined and referred to as AAA hereafter. We identified 19 distinct cell clusters, which is similar to the analyses from original investigators [7,8,9]. Differentially expressed gene (DEG) analysis was performed to identify the marker genes in each cluster. We then assigned cell type identity based on the marker genes and identified major aortic cell types, including three SMC clusters, three fibroblast clusters, two endothelial clusters, pericardial cells, immune cells [four myeloid cells, dendritic cells, B cells, NK&T cells, and neutrophils], and neural cells (Figure 1A). A cluster that expressed low levels of markers expected in cells within the aorta was deemed as cell fragments. Specific gene markers for each cluster are listed in Figure 1B. Consistent with literature findings of SMC loss [2], we found a reduced SMC percentage in all cells recovered in aneurysmal aorta compared to control aorta (29% in TAAs vs. 43% in thoracic aortae and 17.2% in AAAs vs. 36.4% in abdominal aortae; *p* < 0.00001 for both comparisons; Figure 1C).

### 3.2. Phenotypically Modulated SMCs in Thoracic Aortic Aneurysms

**Figure 2:**
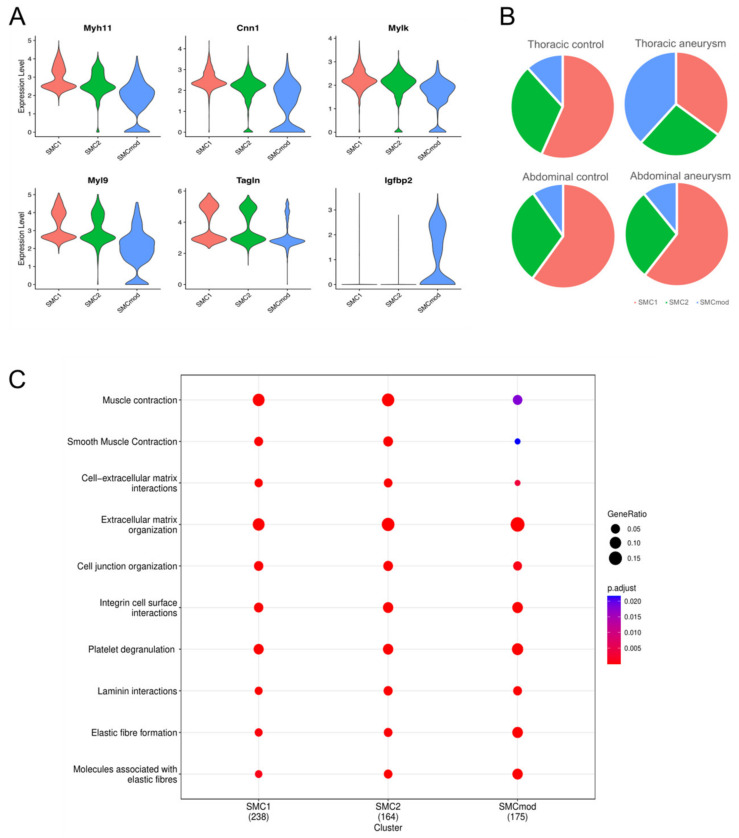
(**A**) Violin plots of smooth muscle cell (SMC) marker genes (*Myh11*, *Cnn1*, *Mylk*, *Myl9*, and *Tagln*) and *Igfbp2* in the three SMC clusters. Note the SMCmod cluster had lower expression of smooth muscle cell markers and higher expression of *Igfbp2*. (**B**) Pie charts showing proportions of the three SMC clusters in different conditions. Note the increased SMCmod proportion (gray) in thoracic aneurysms. (**C**) Enriched Reactomes of the three SMC populations.

Previous analysis of the TAA dataset identified a subset of SMCs that underwent phenotypic modulation; reference-based data integration with an atherosclerosis dataset showed the phenotypically modified TAA SMCs co-segregated with a similar cell cluster in atherosclerosis [7]. Using canonical markers of SMCs (*Cnn1*, *Myh11*, *Mylk*, *Myl9*, and *Tagln*), we identified three SMC clusters in the integrated dataset. While all expressed these SMC markers, one cell cluster consistently expressed the contractile SMC markers at a lower level (Figure 2A). By contrast, this cell population expressed a higher level of *Igfbp2* (Figure 2A), which was a marker of the phenotypically modulated SMCs noted in the Pedroza study [7]. We identified this cluster as the phenotypically modulated SMCs and hereafter refer to it as SMCmod. The percentage of SMCmod in all SMC cells was higher in TAAs compared with other datasets (38.3% vs. 10–12%; *p* < 0.0001 for all pairwise comparisons; Figure 2B). The Reactome pathway enrichment analysis of marker genes revealed all three cell populations were enriched for terms found in smooth muscle cells, including extracellular matrix organization, integrin cell surface interactions, and elastic fiber formation (Figure 2C). By contrast, muscle contraction and smooth muscle contraction were terms enriched in SMC1 and SMC2, but less so in SMCmod (Figure 2C). Comparing SMC1 and SMC2, SMC2 expressed fewer of the mature smooth muscle contraction genes and integrin cell surface interaction genes (Figure S2). SMC2 had a higher expression of genes involved in extracellular matrix organization, post-translational protein phosphorylation, and insulin-like growth factor regulation (Figure S2). Together, these results indicate that thoracic aneurysms harbor a high proportion of a less mature SMC population.

### 3.3. Transcriptional Signatures of TAA and AAA SMCs

**Figure 3:**
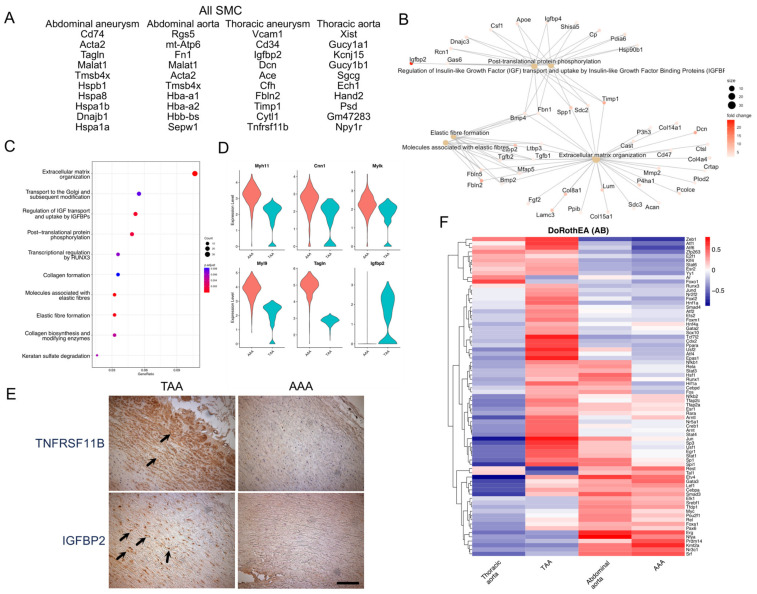
(**A**) Differential expressed gene (DEG) analysis of the top-10 upregulated genes in SMCs from each condition. (**B**) Pathway enrichment analysis showing genes and pathways enriched in TAA SMCs. (**C**) The Reactome analysis showing pathways enriched in TAA SMCs. (**D**) Compared to AAA SMCs, TAA SMCs expressed SMC marker genes at lower levels but had higher *Igfpb2* expression. (**E**) Immunohistochemistry of human TAA (46-year-old male with bicuspid aortic valve-associated aortopathy) and AAA (62-year-old male) tissues demonstrated increased TNFRSF11B and IGFBP2 in TAA SMCs. The lumen was oriented to the upper right corner. (**F**) Regulon analysis predicted transcription factors whose downstream targets were enriched in aortic aneurysms.

The differences in the proportion of cells in smooth muscle cell clusters strongly suggest distinct patterns of SMC phenotypes in TAAs and AAAs. To understand the differences, we computationally combined the smooth muscle cells (SMC1, SMC2, SMCmod; hereafter termed SMC), and compared among conditions (thoracic aneurysms, thoracic controls, abdominal aneurysms, abdominal controls). DEG analysis showed upregulated genes in each condition (Table S1), of which the top-10 genes are listed in Figure 3A. Thoracic aneurysm SMCs showed upregulated *Igfbp2* and *Tnfrsb11b* among 858 upregulated genes and were thoracic aneurysm markers identified in the Pedroza study [7]. Notably, these genes remain upregulated in thoracic aneurysms when compared with abdominal aneurysms. In addition, thoracic aneurysm SMCs upregulate genes relevant in extracellular matrix biology (*Dcn* and *Fbln2*) and *Cd34*, which is a stem cell marker. Pathway enrichment analysis showed that thoracic aneurysm SMCs were enriched for extracellular matrix organization (Figure 3B,C), a finding in keeping for the role of extracellular matrix biology in thoracic aneurysm formation. They are also enriched for “IGF transport and uptake by IGFBPs”, highlighting the specific role of *Igfpb2* in thoracic aneurysms. By contrast, abdominal aneurysm SMCs upregulated 210 genes, with the term “SRP-dependent co-translational protein targeting to membrane” being the most enriched (Figure S3). Thoracic control aortae had more expression of neuronal genes (*Kcnj15* and *Npy1r*), which is consistent with the contribution of a neural crest-derived lineage in the thoracic aortae (Figure 3A). Comparing thoracic and abdominal aneurysm SMCs, abdominal aneurysm SMCs are enriched for smooth muscle marker genes such as *Acta2* and *Tagln*. Comparing smooth muscle cell markers such as *Acta2*, *Tagln*, *Myl9*, *Myl6*, *Cnn1*, and *Myh11*, these markers were expressed less in thoracic aneurysm SMCs (Figure 3D), suggesting a less-differentiated state of the thoracic aneurysm SMCs.

The bioinformatic results strongly suggest TAA and AAA SMCs are transcriptionally distinct entities. We sought to validate these findings in human aortic aneurysms. We obtained formalin-fixed, paraffin-embedded human thoracic aortic aneurysms and abdominal aortic aneurysms. We chose two genes specifically upregulated in TAA SMCs, namely *TNFRSF11B* and *IGFBP2*, for immunohistochemistry (IHC). IHC showed that both TNFRSF11B and IGFBP2 were highly expressed in the SMCs in TAAs, but not AAAs (Figure 3E; representative image shown from n = 3 for AAAs; n = 5 for TAAs), thereby validating the findings from single cell sequencing of mouse aneurysmal models are applicable to human aneurysmal tissues.

To understand the transcriptional machinery underpinning these transcriptional changes, we performed a DoRothEA (Discriminant Regulon Expression Analysis) analysis, which scores the expression of the transcriptional targets of a transcription factor (TF), which is termed a regulon [10]. The expression level of a regulon is a proxy of the transcriptional activity of a given TF. A DoRothEA analysis uses the VIPER method to analyze the enrichment of TF target genes to infer the activity of a certain TFs [10]. This method has been validated for use in analyzing single-cell sequencing datasets [12]. The DoRothEA database includes a curated collection of TF–transcriptional targets, each assigned a confidence score indicating the strength of the supporting evidence. We focused our analysis on TF–transcriptional target relationships of high confidence (confidence level A–B) [10]. We then performed unsupervised clustering to identify the similarity and heterogeneity among SMCs from thoracic and abdominal aortae and aneurysms. A heatmap with unsupervised clustering of regulons on the Y-axis and cell clusters on the X-axis is shown in Figure 3F, which demonstrated thoracic aneurysm SMCs utilize a unique set of TFs and is exemplified by *Ppara* and *Tcf7l2*. Thoracic aneurysm SMCs shared TFs with thoracic aorta SMCs, such as *Zeb1* and *Atf6*, which are not shared with abdominal aorta or aneurysm SMCs, suggesting these TFs may induce anatomic location-specific signals. Thoracic aneurysm SMCs also shared TFs with abdominal aneurysm SMCs, including *Jun* and *Sp3*, which may be signals specific to aneurysm formation. Abdominal aneurysm SMC shared TFs with abdominal aortic SMC including *Erg* and *Nyfa*, which might be abdominal aorta-specific signals. These results demonstrate that aneurysmal SMCs and normal aortic SMCs utilize specific TFs, yet transcriptional similarity exists in SMC from different anatomical locations.

### 3.4. Cell–Cell Interaction Difference Between TAA and AAA SMCs

We next endeavored to understand how gene expression changes in SMCs lead to alterations in cell–cell interaction in thoracic versus abdominal aortic aneurysms. To this aim, we performed connectome analysis [11]. The analysis required the same types of cells as input and a sufficient number cells within each cell type. Hence, we combined the SMC cell clusters, fibroblast clusters, EC clusters, and myeloid clusters. The other cell types were excluded in this analysis due to low cell numbers. We utilized the Connectome R package to generate the ligand–receptor pairs that are upregulated in thoracic aortic aneurysms compared to abdominal aortic aneurysms.

**Figure 4:**
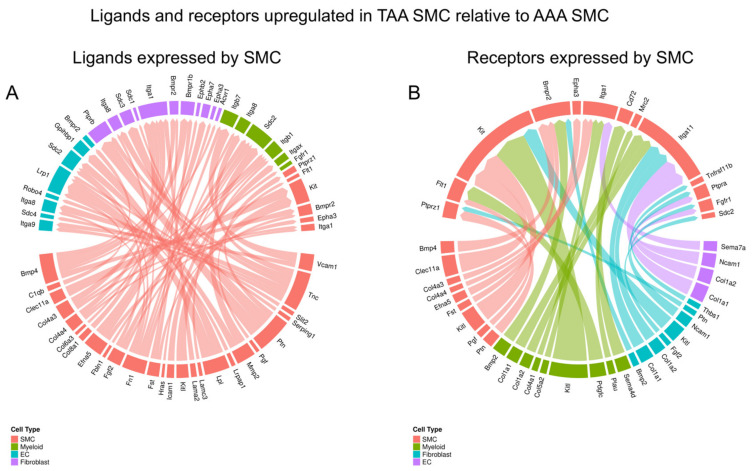
Connectome analysis demonstrated ligand–receptor pairs that were both upregulated in TAA SMCs compared to AAA SMCs, with focus on (**A**) ligands expressed by SMCs and (**B**) receptors expressed by SMCs.

We first focused on ligands expressed in SMCs whereby both ligands and receptors are upregulated in TAAs. As shown in Figure 4A, genes involved in extracellular matrix functions (e.g., *Col4a3*–*Itga1* and *Fn1*–*Itga8*), BMP signaling (*Bmp4*–*Bmpr2* and *Bmp4*–*Bmpr1b*), and FGF signaling (*Fgf2*–*Sdc2*) were among the ligands upregulated in TAA SMCs relative to AAA SMCs. Similarly, BMP signaling (*Bmp4*–*Bmpr2*), ECM functions (e.g., *Thbs1*–*Tnfrsf11b*, *Col4a4*–*Itga1*, and *Col1a1*–*Itga11*) were among the receptors upregulated in TAA SMCs (Figure 4B). These results demonstrate that TAA and AAA SMCs employ different ligand–receptor pairs to interact with themselves and other cell types in the tissue, which may contribute to their clinical and biological differences.

## 4. Discussion

TAAs and AAAs share gross and microscopic morphologic similarities, but have distinct clinical predilection and natural history [2], suggesting different pathophysiological underpinnings. In this meta-analysis of single-cell sequencing of thoracic and abdominal aortic aneurysms and their respective control aortae, we demonstrated distinct patterns of SMC phenotypic modulation in TAAs and AAAs. We found that a phenotypically modulated SMC population is enriched in TAAs but not AAAs or control aortae. TAA SMCs upregulate genes including *Igfbp2* and *Tnfrsf11b*, as well as Reactome terms including extracellular matrix and IGF signaling. Regulon analysis identified transcription factors preferentially used by SMCs from aneurysms. Connectome analysis revealed differential interactions among cell types, with TAAs increasing interactions with extracellular matrix, BMP signaling, and FGF signaling compared to AAAs. Finally, immunostaining on human aortic aneurysms showed increased TNFRSF11B and IGFBP2 proteins in TAAs relative to AAAs, validating the bioinformatic analysis performed on mouse tissues.

SMCs play a central role in the formation of aortic aneurysms [13]. Healthy SMCs sustain a quiescent and contractile phenotype, whereas in various pathogenic stimuli they acquire a synthetic, migratory phenotype. SMC phenotypic plasticity in physiologic and disease states has been extensively studied. SMC phenotypic switching has been demonstrated as an early event in aneurysm formation [14], but the pattern and extent of switching has not been compared between TAAs and AAAs. While there is no single-cell sequencing experiment that directly compares TAAs with AAAs, meta-analysis has recently emerged as an approach to utilize existing datasets for such purposes [15]. Our analysis showed a distinct pattern of SMC phenotypic modulation, whereby the SMCmod cluster was significantly more abundant in TAAs compared to AAAs or control aortae. The SMCmod cluster, characterized by reduced SMC marker gene expression and increased extracellular matrix genes, has been identified in mouse and human TAA single-cell sequencing datasets [7,16]. This finding is unlikely to be just due inter-experiment batch effect as such differences were not found between the two AAA datasets included in this study.

We validated the differential gene expression by immunostaining, demonstrating IGFBP2 protein was increased in TAA SMCs compared to AAA SMCs. IGFBP2 upregulation has been demonstrated in proteomic and transcriptomic studies of human TAAs [4,16,17]. Conversely, IGFBP2 was reduced in AAAs compared to controls [18]. IGFBPs can be circulatory or tissue-bound, where they bind IGF with high affinity and modulate IGF signaling. IGFBP binding may increase the half-life of IGF and hence its bioavailability. Such interaction could also reduce IGF’s potential interaction with receptors. IGFBPs can therefore facilitate or antagonize IGF signaling in a context-specific manner. In addition to transducing IGF signals, IGFBP may have IGF-independent actions [19]. IGFBP2 has motifs for ECM localization [19]. It is perhaps not surprising that altered *IGFBP2* expression is found in animal models of elastinopathy [20] and TAAs that originate from aberrant ECM functions. Despite the bulk of associative evidence, how IGFBP2 mechanistically causes or prevents thoracic and abdominal aneurysm formation will require future studies to elucidate.

Our result indicated increased *TNFRSF11B* in TAA SMCs compared to AAA SMCs. *TNFRSF11B* encodes osteoprotegerin, a soluble glycoprotein of the tumor necrosis factor receptor superfamily. TNFRSF11B functions as a decoy receptor for the receptor activator of nuclear factor-kappa B ligand (RANKL), regulating osteoclast maturation. *TNFRSF11B* is known to play a role in AAA biology. Increased TNFRSF11B is found in the aortae of angiotensin II-stimulated mice (an AAA murine model). *TNFRSF11B*-null mice had increased mortality due to aortic rupture and dissection, a phenotype rescued by supplementation with the TNFRSF11B protein [21], suggesting a protective role of *TNFRSF11B*. Our analysis indicated a higher *TNFRSF11B* expression in TAA SMCs compared to AAA SMCs. Previous single-cell sequencing analyses demonstrated that *TNFRSF11B* marks a phenotypically modulated SMC population in human and murine TAAs [7,16]. The role of *TNFRSF11B* in TAAs relative to AAAs awaits further investigation.

SMC is a versatile cell type that can exhibit multiple phenotypes in response to physiologic and pathologic stimuli [22]. The plasticity of SMC phenotypes depends on the complex combinatorial interactions between multiple transcription factors [23]. Our regulon analysis revealed a distinct transcription factor signature of TAA SMCs, and to a lesser degree AAA SMCs compared to control aortae. Many regulons identified in this analysis have been demonstrated to have critical functions in aortic aneurysms. For example, the regulon of *TCF7L2* was highly upregulated by TAA SMCs in our study. Genome-wide association studies (GWAS) have identified regulatory *TCF7L2* genetic variants associated with TAAs [24]. Similarly, *ERG*, whose regulon is highly upregulated in AAA SMCs, has been described in GWAS to associate with AAAs [25]. These results indicate that regulon analysis identifies known, important TF networks in aortic aneurysms. It is likely the novel TFs identified in this analysis play relevant roles in aneurysm formation. The functional significance of these novel regulons and how they integrate into gene regulatory networks in aneurysm formation will require future studies.

Our findings provide insights into the literature describing aortic aneurysm single-cell sequencing experiments [7,8,9]. While these studies showed transcriptional differences between aneurysmal and normal aortic SMCs, our analysis unequivocally demonstrated a distinct transcriptional program difference between TAA and AAA SMCs. Specifically, the *Igfbp2*- and *Tnfrsf11b*-expressing modSMC cells were over-represented in TAA SMCs, which is a finding we validated by immunostaining of human pathological samples. Our bioinformatic analysis provided Connectome and Reactome terms that might inform future studies to discover potential therapeutic targets for these pathophysiologically distinct conditions.

Some limitations exist for our analysis. Since the included studies used mice of different ages and sex (see Section 2), this could contribute to the observed findings. Batch effect might affect comparison of datasets generated in different labs at different times, despite our best practice in correction. We included two mouse AAA surgical models, whereas data from the *Fbn1^C1041G/+^* model were included to represent TAAs. Other TAA mouse genetic models exist (e.g., *Fbln4^SMKO^* [26] and *Fbln1^mgR^* [27]). However, no signal-cell sequencing datasets were available for these models, thereby limiting our ability to include these alternative animal models.

Part of this research had been presented in 2023 American College of Cardiology Annual Scientific Sessions as a poster abstract [28].

## Figures and Tables

**Table 1:** Datasets included in this study.

Tissue	TAA	AAA	AAA
Study	Pedroza [7]	Zhao [8]	Yang [9]
GEO accession	GSE153534	GSE152583	GSE164678
Model	*Fbn1^C1041G/+^*	Elastase 14 days	CaCl_2_ 4 days
Control age	24 weeks	10 weeks	12 weeks
Gender	Mixed in control, male in aneurysm	Male	Male
Isolation method	Enzymatic	Enzymatic	Enzymatic
Platform	10X Chromium	10X Chromium	10X Chromium
Number of cells (aneurysm/control)	3765/2823	1400/1392	1216/2035

**Table 2:** Human aortic aneurysm samples included in this study.

Tissue	TAA	AAA
Demographics	21-year-old male, Marfan syndrome 22-year-old male, Marfan syndrome 36-year-old male, Marfan syndrome 65-year-old male, nonsyndromic 46-year-old male, bicuspid aortic valve	52-year-old male 72-year-old female 62-year-old male

## Data Availability

The code used for the analysis is available upon request. The single cell sequencing datasets can be downloaded from the Gene Expression Omnibus, with accession number indicated in Table 1.

## Supplementary Materials

The following supporting information can be downloaded at: https://www.mdpi.com/article/10.3390/jcdd11110349/s1, Figure S1: CaCl_2_ and elastase treatment yielded similar cell type distributions in mouse AAA models; Figure S2: (A) Reactome enrichment of SMC1 compared to SMC2. (B) Enriched Reactome terms and corresponding genes in SMC1 compared to SMC2. (C) Enriched Reactome terms enriched in SMC2 compared to SMC1. Figure S3: Reactome terms (A) and genes (B) enriched in abdominal aortic aneurysm SMCs compared to thoracic aortic aneurysm SMCs.; Table S1: Upregulated genes in SMC of control and aneurysmal thoracic and abbominal aortae.

## Abbreviations

TAA: thoracic aortic aneurysm; AAA, abdominal aortic aneurysm; SMC, smooth muscle cell; IGFBP2, insulin-like growth factor binding protein 2; UMAP, uniform manifold approximation and projection; DEG, differentially expressed genes.
