# Supplementary material for: Distinct Patterns of Smooth Muscle Phenotypic Modulation in Thoracic and Abdominal Aortic Aneurysms
Source: J Cardiovasc Dev Dis. 2024 Nov 1;11(11):349. doi: 10.3390/jcdd11110349 (PMC11594343; doi:10.3390/jcdd11110349)
Supplement: Supplementary file 1 [file jcdd-11-00349-s001.zip › jcdd-3205829 supplemtary figures.pdf]

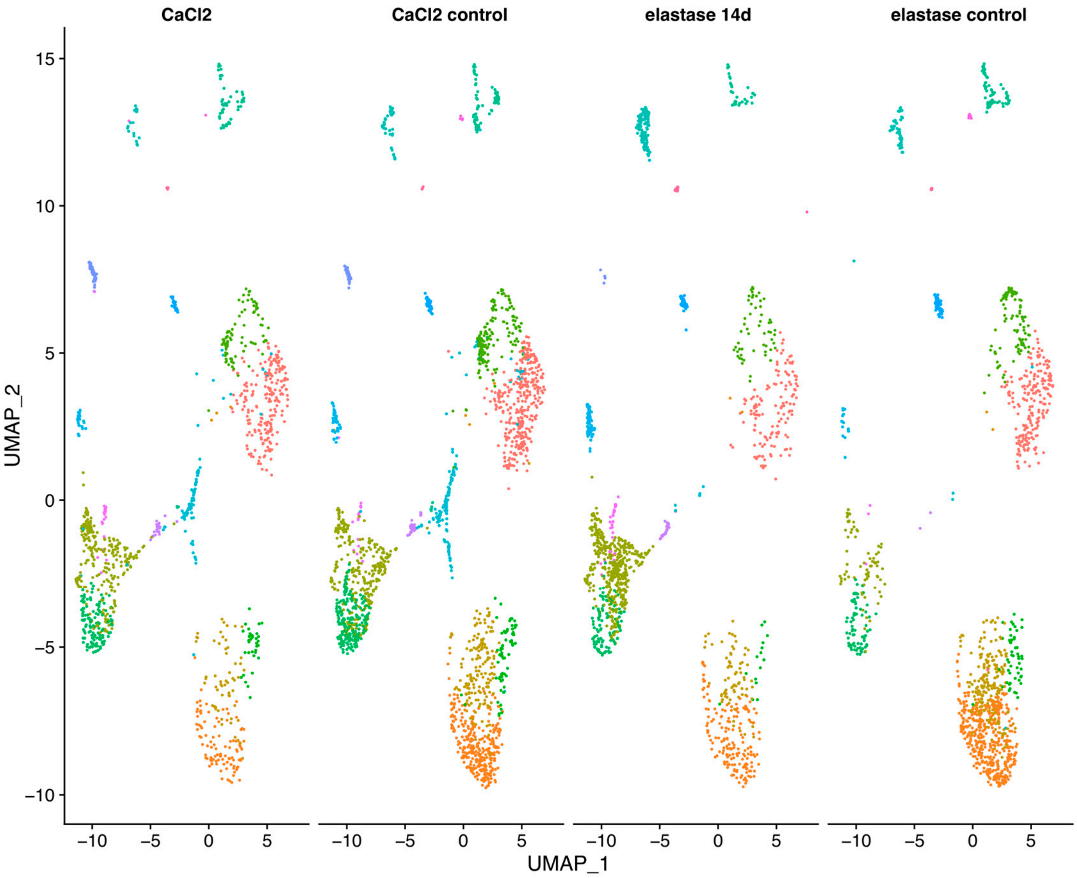

Figure S1. CaCl2 and elastase treatment yielded similar cell type distributions in mouse AAA models.

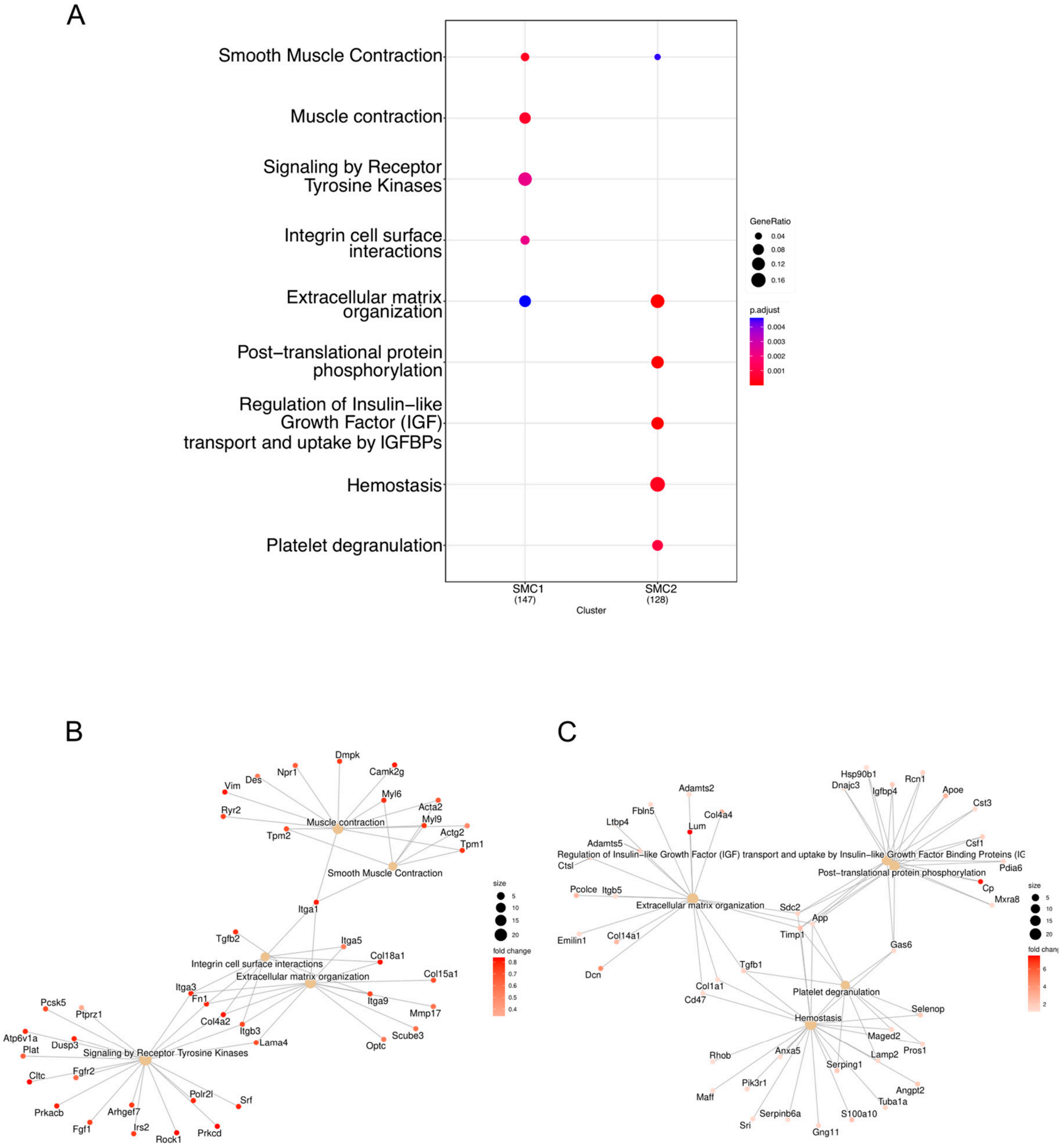

Figure S2. (A) Reactome enrichment of SMC1 compared to SMC2. (B) Enriched Reactome terms and corresponding genes in SMC1 compared to SMC2. (C) Enriched Reactome terms enriched in SMC2 compared to SMC1.

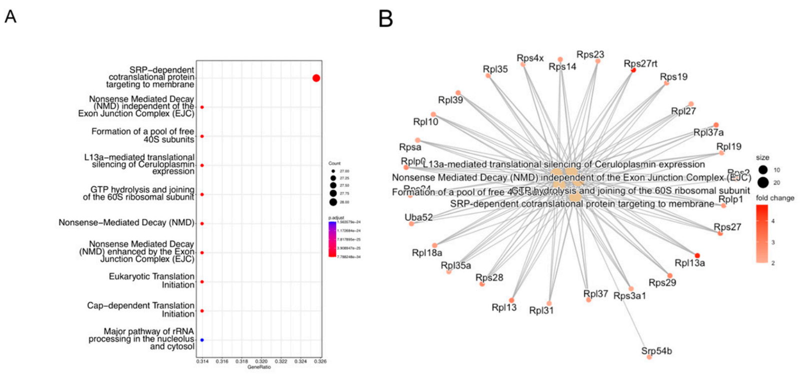

Figure S3. Reactome terms (A) and genes (B) enriched in abdominal aortic aneurysm SMCs compared to thoracic aortic aneurysm SMCs.
